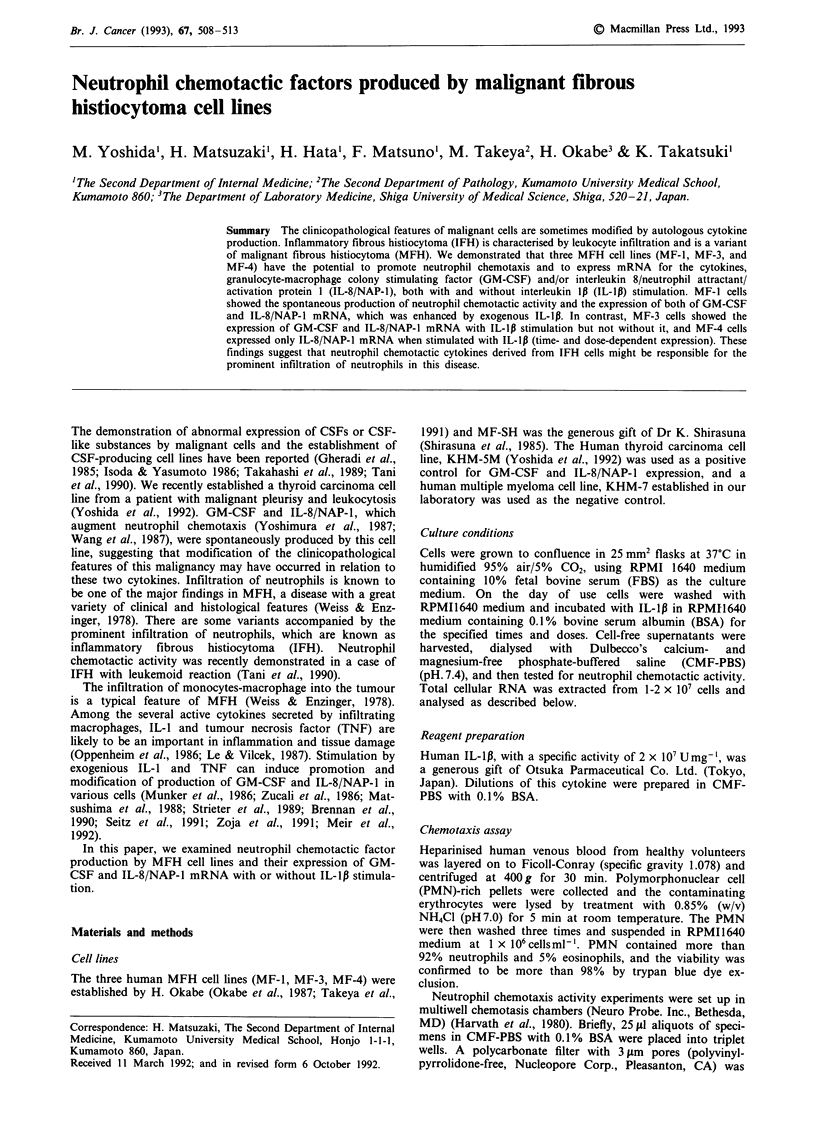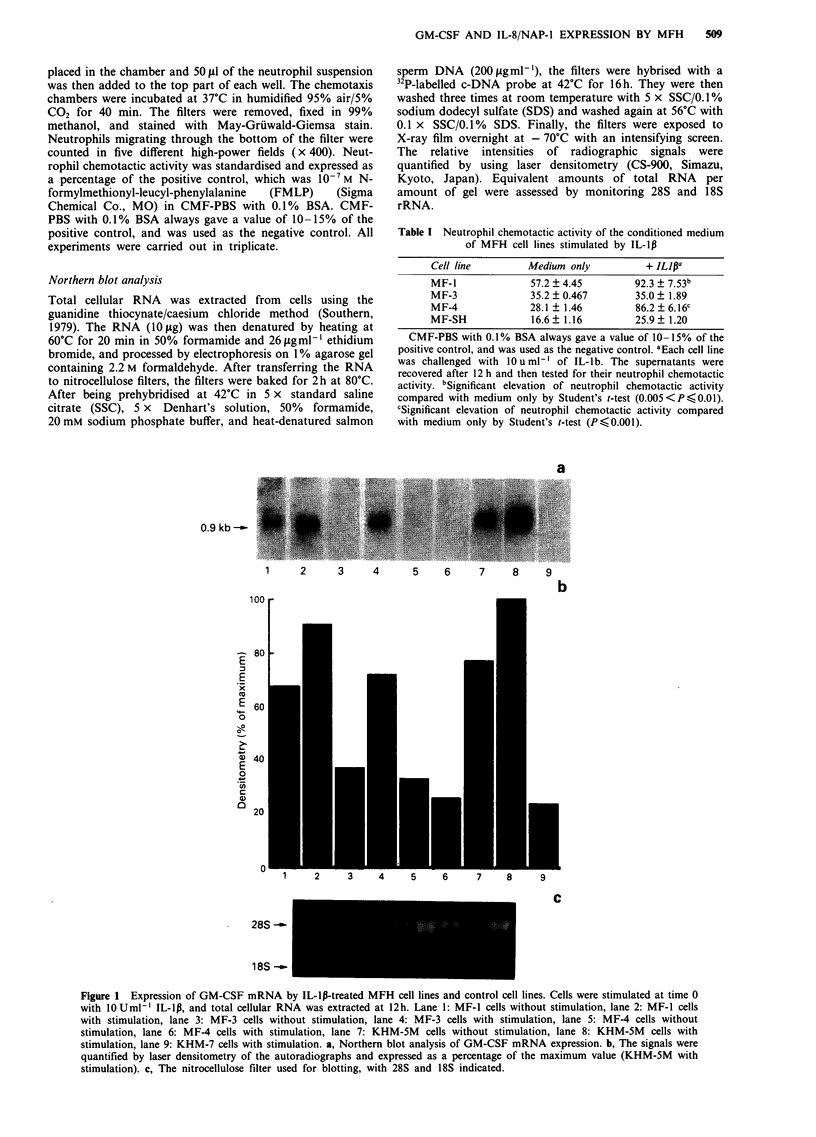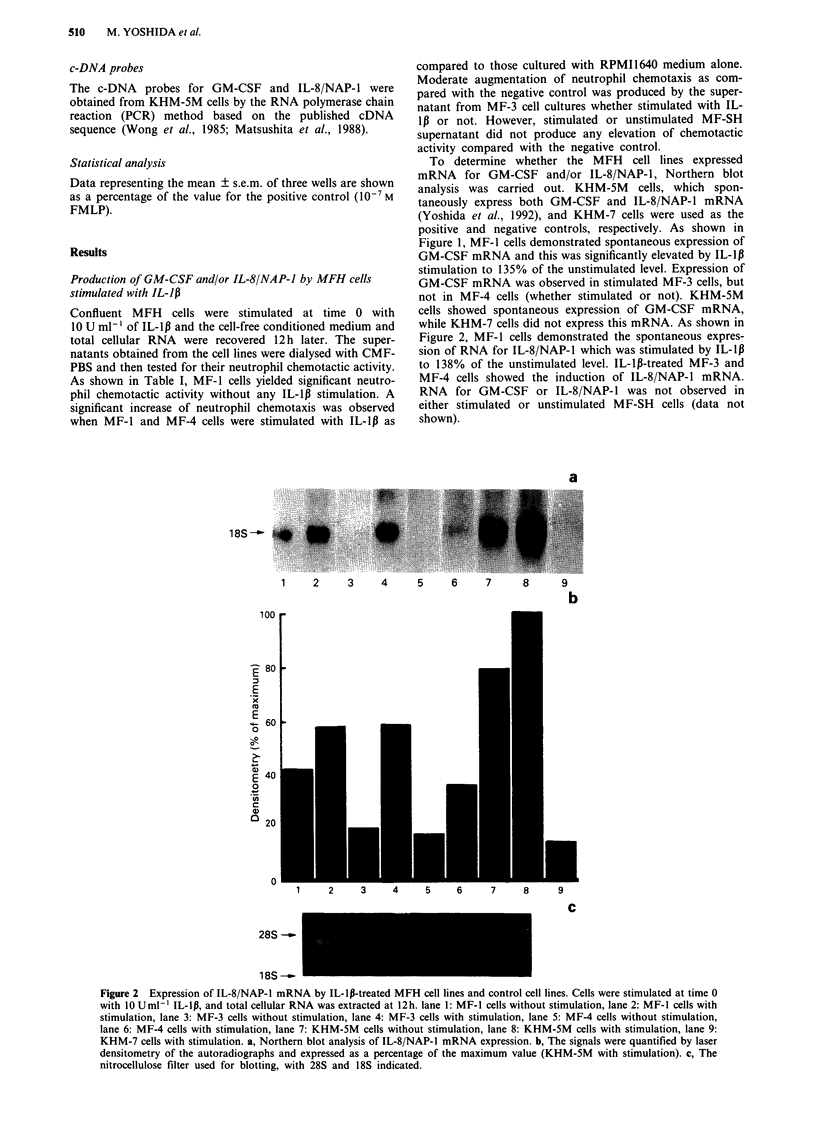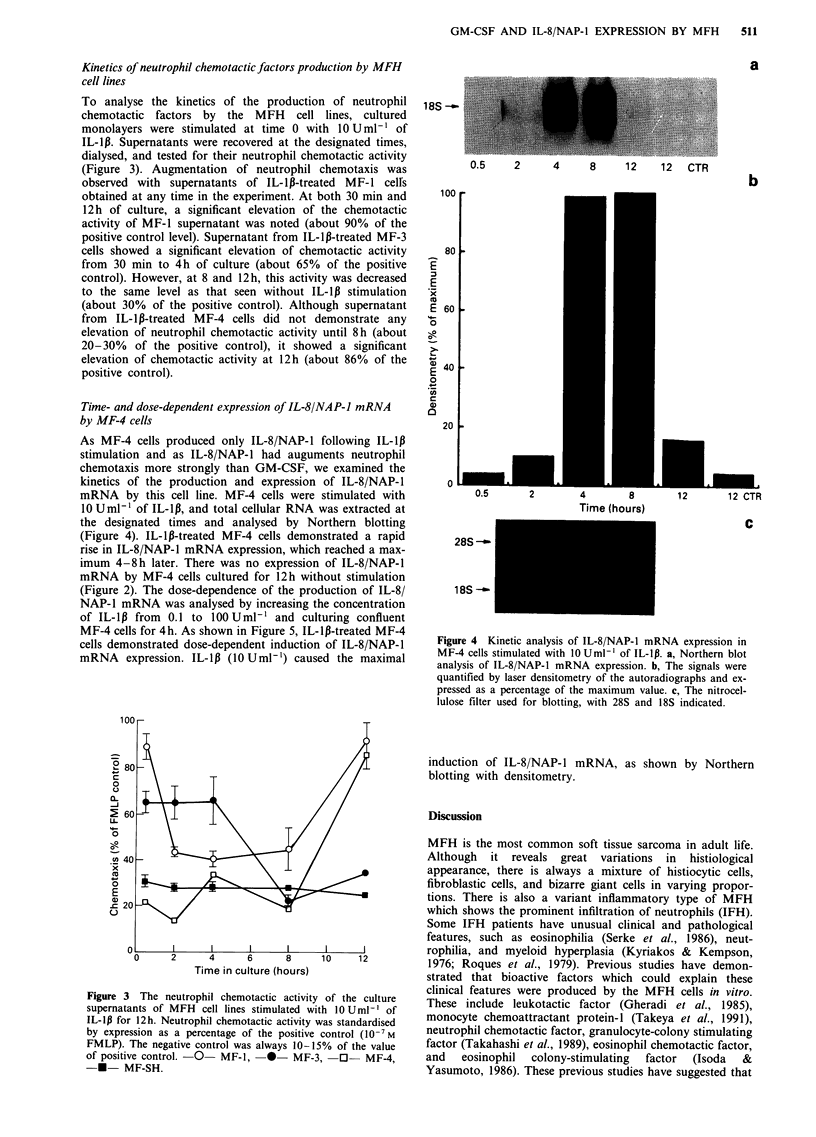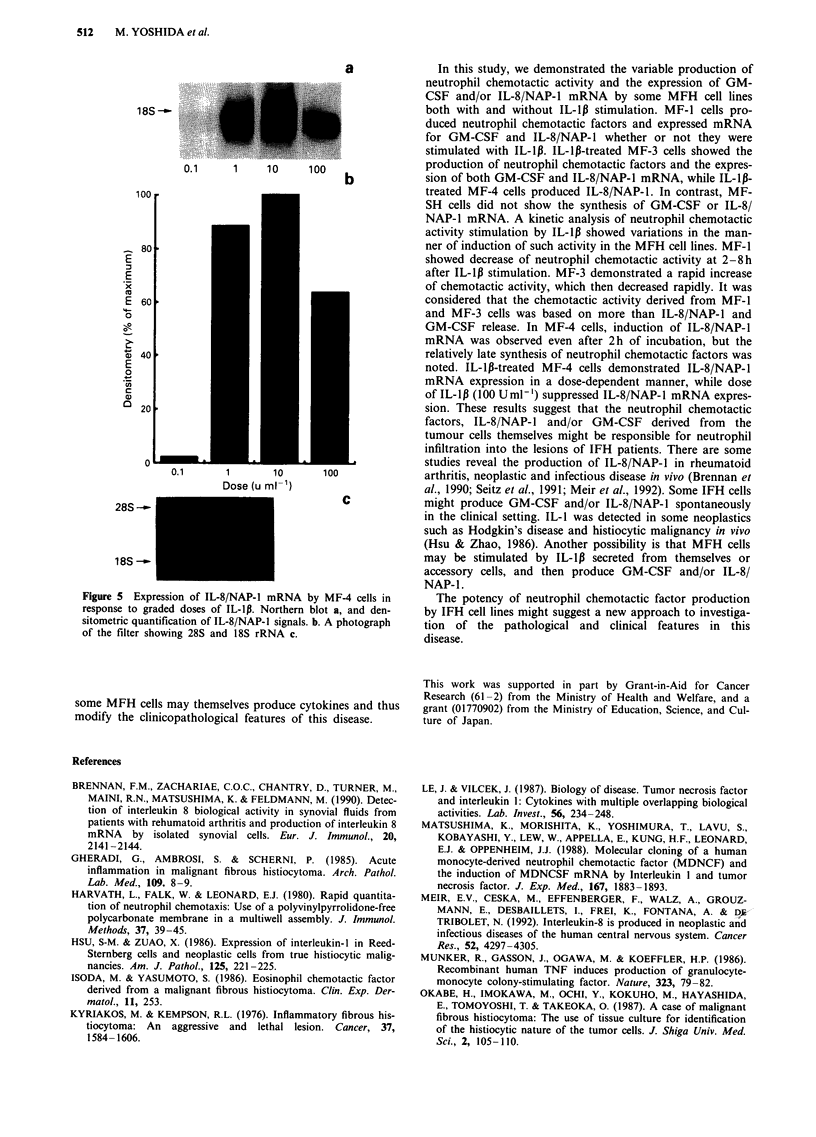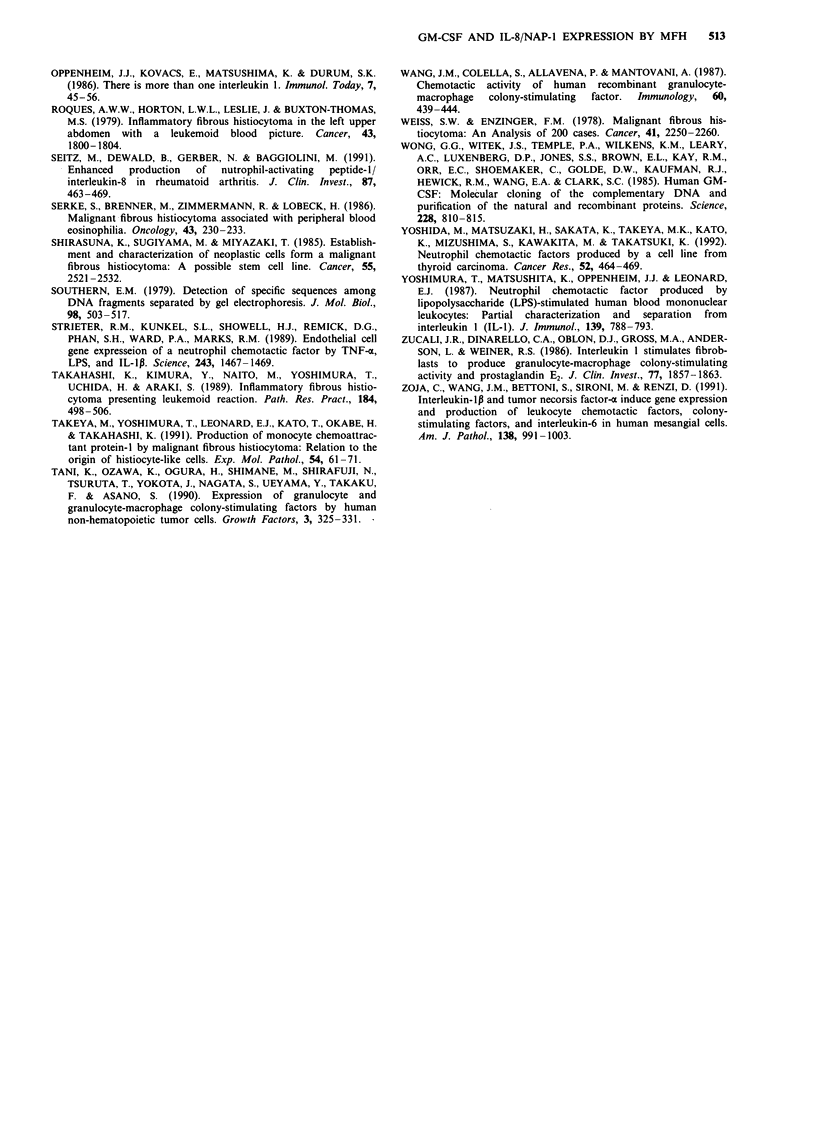# Neutrophil chemotactic factors produced by malignant fibrous histiocytoma cell lines.

**DOI:** 10.1038/bjc.1993.95

**Published:** 1993-03

**Authors:** M. Yoshida, H. Matsuzaki, H. Hata, F. Matsuno, M. Takeya, H. Okabe, K. Takatsuki

**Affiliations:** Second Department of Internal Medicine, Kumamoto University Medical School, Japan.

## Abstract

**Images:**


*Br. J. Cancer* (1993), **67**, 508–513 © Macmillan Press Ltd., 1993

### Neutrophil chemotactic factors produced by malignant fibrous histiocytoma cell lines

M. Yoshida[1], H. Matsuzaki[1], H. Hata[1], F. Matsuno[1], M. Takeya[2], H. Okabe[3] & K. Takatsuki[1]

*[1]The Second Department of Internal Medicine; [2]The Second Department of Pathology, Kumamoto University Medical School, Kumamoto 860; [3]The Department of Laboratory Medicine, Shiga University of Medical Science, Shiga, 520–21, Japan.*

**Summary** The clinicopathological features of malignant cells are sometimes modified by autologous cytokine production. Inflammatory fibrous histiocytoma (IFH) is characterised by leukocyte infiltration and is a variant of malignant fibrous histiocytoma (MFH). We demonstrated that three MFH cell lines (MF-1, MF-3, and MF-4) have the potential to promote neutrophil chemotaxis and to express mRNA for the cytokines, granulocyte-macrophage colony stimulating factor (GM-CSF) and/or interleukin 8/neutrophil attractant/activation protein 1 (IL-8/NAP-1), both with and without interleukin 1β (IL-1β) stimulation. MF-1 cells showed the spontaneous production of neutrophil chemotactic activity and the expression of both of GM-CSF and IL-8/NAP-1 mRNA, which was enhanced by exogenous IL-1β. In contrast, MF-3 cells showed the expression of GM-CSF and IL-8/NAP-1 mRNA with IL-1β stimulation but not without it, and MF-4 cells expressed only IL-8/NAP-1 mRNA when stimulated with IL-1β (time- and dose-dependent expression). These findings suggest that neutrophil chemotactic cytokines derived from IFH cells might be responsible for the prominent infiltration of neutrophils in this disease.

The demonstration of abnormal expression of CSFs or CSF-like substances by malignant cells and the establishment of CSF-producing cell lines have been reported (Gheradi *et al.*, 1985; Isoda & Yasumoto 1986; Takahashi *et al.*, 1989; Tani *et al.*, 1990). We recently established a thyroid carcinoma cell line from a patient with malignant pleurisy and leukocytosis (Yoshida *et al.*, 1992). GM-CSF and IL-8/NAP-1, which augment neutrophil chemotaxis (Yoshimura *et al.*, 1987; Wang *et al.*, 1987), were spontaneously produced by this cell line, suggesting that modification of the clinicopathological features of this malignancy may have occurred in relation to these two cytokines. Infiltration of neutrophils is known to be one of the major findings in MFH, a disease with a great variety of clinical and histological features (Weiss & Enzinger, 1978). There are some variants accompanied by the prominent infiltration of neutrophils, which are known as inflammatory fibrous histiocytoma (IFH). Neutrophil chemotactic activity was recently demonstrated in a case of IFH with leukemoid reaction (Tani *et al.*, 1990).

The infiltration of monocytes-macrophage into the tumour is a typical feature of MFH (Weiss & Enzinger, 1978). Among the several active cytokines secreted by infiltrating macrophages, IL-1 and tumour necrosis factor (TNF) are likely to be an important in inflammation and tissue damage (Oppenheim *et al.*, 1986; Le & Vilcek, 1987). Stimulation by exogenious IL-1 and TNF can induce promotion and modification of production of GM-CSF and IL-8/NAP-1 in various cells (Munker *et al.*, 1986; Zucali *et al.*, 1986; Matsushima *et al.*, 1988; Strieter *et al.*, 1989; Brennan *et al.*, 1990; Seitz *et al.*, 1991; Zoja *et al.*, 1991; Meir *et al.*, 1992).

In this paper, we examined neutrophil chemotactic factor production by MFH cell lines and their expression of GM-CSF and IL-8/NAP-1 mRNA with or without IL-1β stimulation.

### Materials and methods

### *Cell lines*

The three human MFH cell lines (MF-1, MF-3, MF-4) were established by H. Okabe (Okabe *et al.*, 1987; Takeya *et al.*, 1991) and MF-SH was the generous gift of Dr K. Shirasuna (Shirasuna *et al.*, 1985). The Human thyroid carcinoma cell line, KHM-5M (Yoshida *et al.*, 1992) was used as a positive control for GM-CSF and IL-8/NAP-1 expression, and a human multiple myeloma cell line, KHM-7 established in our laboratory was used as the negative control.

### *Culture conditions*

Cells were grown to confluence in 25 $mm^2$ flasks at 37°C in humidified 95% air/5% $CO_2$, using RPMI 1640 medium containing 10% fetal bovine serum (FBS) as the culture medium. On the day of use cells were washed with RPMI1640 medium and incubated with IL-1β in RPMI1640 medium containing 0.1% bovine serum albumin (BSA) for the specified times and doses. Cell-free supernatants were harvested, dialysed with Dulbecco's calcium- and magnesium-free phosphate-buffered saline (CMF-PBS) (pH. 7.4), and then tested for neutrophil chemotactic activity. Total cellular RNA was extracted from $1\text{-}2 \times 10^7$ cells and analysed as described below.

### *Reagent preparation*

Human IL-1β, with a specific activity of $2 \times 10^7$ U $mg^{-1}$, was a generous gift of Otsuka Parmaceutical Co. Ltd. (Tokyo, Japan). Dilutions of this cytokine were prepared in CMF-PBS with 0.1% BSA.

### *Chemotaxis assay*

Heparinised human venous blood from healthy volunteers was layered on to Ficoll-Conray (specific gravity 1.078) and centrifuged at 400 *g* for 30 min. Polymorphonuclear cell (PMN)-rich pellets were collected and the contaminating erythrocytes were lysed by treatment with 0.85% (w/v) $NH_4Cl$ (pH 7.0) for 5 min at room temperature. The PMN were then washed three times and suspended in RPMI1640 medium at $1 \times 10^6$ cells $ml^{-1}$. PMN contained more than 92% neutrophils and 5% eosinophils, and the viability was confirmed to be more than 98% by trypan blue dye exclusion.

Neutrophil chemotaxis activity experiments were set up in multiwell chemotasis chambers (Neuro Probe. Inc., Bethesda, MD) (Harvath *et al.*, 1980). Briefly, 25 μl aliquots of specimens in CMF-PBS with 0.1% BSA were placed into triplet wells. A polycarbonate filter with 3 μm pores (polyvinylpyrrolidone-free, Nucleopore Corp., Pleasanton, CA) was

Correspondence: H. Matsuzaki, The Second Department of Internal Medicine, Kumamoto University Medical School, Honjo 1-1-1, Kumamoto 860, Japan.
Received 11 March 1992; and in revised form 6 October 1992.

placed in the chamber and 50 μl of the neutrophil suspension was then added to the top part of each well. The chemotaxis chambers were incubated at 37°C in humidified 95% air/5% $CO_2$ for 40 min. The filters were removed, fixed in 99% methanol, and stained with May-Grüwald-Giemsa stain. Neutrophils migrating through the bottom of the filter were counted in five different high-power fields (× 400). Neutrophil chemotactic activity was standardised and expressed as a percentage of the positive control, which was $10^{-7}$ M N-formylmethionyl-leucyl-phenylalanine (FMLP) (Sigma Chemical Co., MO) in CMF-PBS with 0.1% BSA. CMF-PBS with 0.1% BSA always gave a value of 10–15% of the positive control, and was used as the negative control. All experiments were carried out in triplicate.

*Northern blot analysis*

Total cellular RNA was extracted from cells using the guanidine thiocyanate/caesium chloride method (Southern, 1979). The RNA (10 μg) was then denatured by heating at 60°C for 20 min in 50% formamide and 26 $\mu g ml^{-1}$ ethidium bromide, and processed by electrophoresis on 1% agarose gel containing 2.2 M formaldehyde. After transferring the RNA to nitrocellulose filters, the filters were baked for 2 h at 80°C. After being prehybridised at 42°C in 5 × standard saline citrate (SSC), 5 × Denhart's solution, 50% formamide, 20 mM sodium phosphate buffer, and heat-denatured salmon sperm DNA (200 $\mu g ml^{-1}$), the filters were hybrised with a $^{32}P$-labelled c-DNA probe at 42°C for 16 h. They were then washed three times at room temperature with 5 × SSC/0.1% sodium dodecyl sulfate (SDS) and washed again at 56°C with 0.1 × SSC/0.1% SDS. Finally, the filters were exposed to X-ray film overnight at – 70°C with an intensifying screen. The relative intensities of radiographic signals were quantified by using laser densitometry (CS-900, Simazu, Kyoto, Japan). Equivalent amounts of total RNA per amount of gel were assessed by monitoring 28S and 18S rRNA.

**Table I** Neutrophil chemotactic activity of the conditioned medium of MFH cell lines stimulated by IL-1β

| *Cell line* | *Medium only* | *+ IL1β*[a] |
|---|---|---|
| MF-1 | 57.2 ± 4.45 | 92.3 ± 7.53[b] |
| MF-3 | 35.2 ± 0.467 | 35.0 ± 1.89 |
| MF-4 | 28.1 ± 1.46 | 86.2 ± 6.16[c] |
| MF-SH | 16.6 ± 1.16 | 25.9 ± 1.20 |

CMF-PBS with 0.1% BSA always gave a value of 10–15% of the positive control, and was used as the negative control. [a]Each cell line was challenged with 10 $u ml^{-1}$ of IL-1b. The supernatants were recovered after 12 h and then tested for their neutrophil chemotactic activity. [b]Significant elevation of neutrophil chemotactic activity compared with medium only by Student's *t*-test ($0.005 < P \leqslant 0.01$). [c]Significant elevation of neutrophil chemotactic activity compared with medium only by Student's *t*-test ($P \leqslant 0.001$).

**Figure 1** Expression of GM-CSF mRNA by IL-1β-treated MFH cell lines and control cell lines. Cells were stimulated at time 0 with 10 $U ml^{-1}$ IL-1β, and total cellular RNA was extracted at 12h. Lane 1: MF-1 cells without stimulation, lane 2: MF-1 cells with stimulation, lane 3: MF-3 cells without stimulation, lane 4: MF-3 cells with stimulation, lane 5: MF-4 cells without stimulation, lane 6: MF-4 cells with stimulation, lane 7: KHM-5M cells without stimulation, lane 8: KHM-5M cells with stimulation, lane 9: KHM-7 cells with stimulation. **a**, Northern blot analysis of GM-CSF mRNA expression. **b**, The signals were quantified by laser densitometry of the autoradiographs and expressed as a percentage of the maximum value (KHM-5M with stimulation). **c**, The nitrocellulose filter used for blotting, with 28S and 18S indicated.

### c-DNA probes

The c-DNA probes for GM-CSF and IL-8/NAP-1 were obtained from KHM-5M cells by the RNA polymerase chain reaction (PCR) method based on the published cDNA sequence (Wong *et al.*, 1985; Matsushita *et al.*, 1988).

### Statistical analysis

Data representing the mean ± s.e.m. of three wells are shown as a percentage of the value for the positive control ($10^{-7}$ M FMLP).

### Results

### Production of GM-CSF and/or IL-8/NAP-1 by MFH cells stimulated with IL-1β

Confluent MFH cells were stimulated at time 0 with $10\ U\ ml^{-1}$ of IL-1β and the cell-free conditioned medium and total cellular RNA were recovered 12h later. The supernatants obtained from the cell lines were dialysed with CMF-PBS and then tested for their neutrophil chemotactic activity. As shown in Table I, MF-1 cells yielded significant neutrophil chemotactic activity without any IL-1β stimulation. A significant increase of neutrophil chemotaxis was observed when MF-1 and MF-4 cells were stimulated with IL-1β as compared to those cultured with RPMI1640 medium alone. Moderate augmentation of neutrophil chemotaxis as compared with the negative control was produced by the supernatant from MF-3 cell cultures whether stimulated with IL-1β or not. However, stimulated or unstimulated MF-SH supernatant did not produce any elevation of chemotactic activity compared with the negative control.

To determine whether the MFH cell lines expressed mRNA for GM-CSF and/or IL-8/NAP-1, Northern blot analysis was carried out. KHM-5M cells, which spontaneously express both GM-CSF and IL-8/NAP-1 mRNA (Yoshida *et al.*, 1992), and KHM-7 cells were used as the positive and negative controls, respectively. As shown in Figure 1, MF-1 cells demonstrated spontaneous expression of GM-CSF mRNA and this was significantly elevated by IL-1β stimulation to 135% of the unstimulated level. Expression of GM-CSF mRNA was observed in stimulated MF-3 cells, but not in MF-4 cells (whether stimulated or not). KHM-5M cells showed spontaneous expression of GM-CSF mRNA, while KHM-7 cells did not express this mRNA. As shown in Figure 2, MF-1 cells demonstrated the spontaneous expression of RNA for IL-8/NAP-1 which was stimulated by IL-1β to 138% of the unstimulated level. IL-1β-treated MF-3 and MF-4 cells showed the induction of IL-8/NAP-1 mRNA. RNA for GM-CSF or IL-8/NAP-1 was not observed in either stimulated or unstimulated MF-SH cells (data not shown).

**Figure 2** Expression of IL-8/NAP-1 mRNA by IL-1β-treated MFH cell lines and control cell lines. Cells were stimulated at time 0 with $10\ U ml^{-1}$ IL-1β, and total cellular RNA was extracted at 12h. lane 1: MF-1 cells without stimulation, lane 2: MF-1 cells with stimulation, lane 3: MF-3 cells without stimulation, lane 4: MF-3 cells with stimulation, lane 5: MF-4 cells without stimulation, lane 6: MF-4 cells with stimulation, lane 7: KHM-5M cells without stimulation, lane 8: KHM-5M cells with stimulation, lane 9: KHM-7 cells with stimulation. **a**, Northern blot analysis of IL-8/NAP-1 mRNA expression. **b**, The signals were quantified by laser densitometry of the autoradiographs and expressed as a percentage of the maximum value (KHM-5M with stimulation). **c**, The nitrocellulose filter used for blotting, with 28S and 18S indicated.

*Kinetics of neutrophil chemotactic factors production by MFH cell lines*

To analyse the kinetics of the production of neutrophil chemotactic factors by the MFH cell lines, cultured monolayers were stimulated at time 0 with 10 U ml$^{-1}$ of IL-1β. Supernatants were recovered at the designated times, dialysed, and tested for their neutrophil chemotactic activity (Figure 3). Augmentation of neutrophil chemotaxis was observed with supernatants of IL-1β-treated MF-1 cells obtained at any time in the experiment. At both 30 min and 12 h of culture, a significant elevation of the chemotactic activity of MF-1 supernatant was noted (about 90% of the positive control level). Supernatant from IL-1β-treated MF-3 cells showed a significant elevation of chemotactic activity from 30 min to 4 h of culture (about 65% of the positive control). However, at 8 and 12 h, this activity was decreased to the same level as that seen without IL-1β stimulation (about 30% of the positive control). Although supernatant from IL-1β-treated MF-4 cells did not demonstrate any elevation of neutrophil chemotactic activity until 8 h (about 20–30% of the positive control), it showed a significant elevation of chemotactic activity at 12 h (about 86% of the positive control).

*Time- and dose-dependent expression of IL-8/NAP-1 mRNA by MF-4 cells*

As MF-4 cells produced only IL-8/NAP-1 following IL-1β stimulation and as IL-8/NAP-1 had auguments neutrophil chemotaxis more strongly than GM-CSF, we examined the kinetics of the production and expression of IL-8/NAP-1 mRNA by this cell line. MF-4 cells were stimulated with 10 U ml$^{-1}$ of IL-1β, and total cellular RNA was extracted at the designated times and analysed by Northern blotting (Figure 4). IL-1β-treated MF-4 cells demonstrated a rapid rise in IL-8/NAP-1 mRNA expression, which reached a maximum 4–8 h later. There was no expression of IL-8/NAP-1 mRNA by MF-4 cells cultured for 12 h without stimulation (Figure 2). The dose-dependence of the production of IL-8/NAP-1 mRNA was analysed by increasing the concentration of IL-1β from 0.1 to 100 U ml$^{-1}$ and culturing confluent MF-4 cells for 4 h. As shown in Figure 5, IL-1β-treated MF-4 cells demonstrated dose-dependent induction of IL-8/NAP-1 mRNA expression. IL-1β (10 U ml$^{-1}$) caused the maximal induction of IL-8/NAP-1 mRNA, as shown by Northern blotting with densitometry.

Figure 4 Kinetic analysis of IL-8/NAP-1 mRNA expression in MF-4 cells stimulated with 10 U ml$^{-1}$ of IL-1β. **a**, Northern blot analysis of IL-8/NAP-1 mRNA expression. **b**, The signals were quantified by laser densitometry of the autoradiographs and expressed as a percentage of the maximum value. **c**, The nitrocellulose filter used for blotting, with 28S and 18S indicated.

**Figure 3** The neutrophil chemotactic activity of the culture supernatants of MFH cell lines stimulated with 10 U ml$^{-1}$ of IL-1β for 12 h. Neutrophil chemotactic activity was standardised by expression as a percentage of the positive control ($10^{-7}$ M FMLP). The negative control was always 10–15% of the value of positive control. —○— MF-1, —●— MF-3, —□— MF-4, —■— MF-SH.

### Discussion

MFH is the most common soft tissue sarcoma in adult life. Although it reveals great variations in histiological appearance, there is always a mixture of histiocytic cells, fibroblastic cells, and bizarre giant cells in varying proportions. There is also a variant inflammatory type of MFH which shows the prominent infiltration of neutrophils (IFH). Some IFH patients have unusual clinical and pathological features, such as eosinophilia (Serke *et al.*, 1986), neutrophilia, and myeloid hyperplasia (Kyriakos & Kempson, 1976; Roques *et al.*, 1979). Previous studies have demonstrated that bioactive factors which could explain these clinical features were produced by the MFH cells *in vitro.* These include leukotactic factor (Gheradi *et al.*, 1985), monocyte chemoattractant protein-1 (Takeya *et al.*, 1991), neutrophil chemotactic factor, granulocyte-colony stimulating factor (Takahashi *et al.*, 1989), eosinophil chemotactic factor, and eosinophil colony-stimulating factor (Isoda & Yasumoto, 1986). These previous studies have suggested that

**Figure 5** Expression of IL-8/NAP-1 mRNA by MF-4 cells in response to graded doses of IL-1β. Northern blot **a**, and densitometric quantification of IL-8/NAP-1 signals. **b**. A photograph of the filter showing 28S and 18S rRNA **c**.

some MFH cells may themselves produce cytokines and thus modify the clinicopathological features of this disease.

In this study, we demonstrated the variable production of neutrophil chemotactic activity and the expression of GM-CSF and/or IL-8/NAP-1 mRNA by some MFH cell lines both with and without IL-1β stimulation. MF-1 cells produced neutrophil chemotactic factors and expressed mRNA for GM-CSF and IL-8/NAP-1 whether or not they were stimulated with IL-1β. IL-1β-treated MF-3 cells showed the production of neutrophil chemotactic factors and the expression of both GM-CSF and IL-8/NAP-1 mRNA, while IL-1β-treated MF-4 cells produced IL-8/NAP-1. In contrast, MF-SH cells did not show the synthesis of GM-CSF or IL-8/NAP-1 mRNA. A kinetic analysis of neutrophil chemotactic activity stimulation by IL-1β showed variations in the manner of induction of such activity in the MFH cell lines. MF-1 showed decrease of neutrophil chemotactic activity at 2–8 h after IL-1β stimulation. MF-3 demonstrated a rapid increase of chemotactic activity, which then decreased rapidly. It was considered that the chemotactic activity derived from MF-1 and MF-3 cells was based on more than IL-8/NAP-1 and GM-CSF release. In MF-4 cells, induction of IL-8/NAP-1 mRNA was observed even after 2 h of incubation, but the relatively late synthesis of neutrophil chemotactic factors was noted. IL-1β-treated MF-4 cells demonstrated IL-8/NAP-1 mRNA expression in a dose-dependent manner, while dose of IL-1β (100 U ml$^{-1}$) suppressed IL-8/NAP-1 mRNA expression. These results suggest that the neutrophil chemotactic factors, IL-8/NAP-1 and/or GM-CSF derived from the tumour cells themselves might be responsible for neutrophil infiltration into the lesions of IFH patients. There are some studies reveal the production of IL-8/NAP-1 in rheumatoid arthritis, neoplastic and infectious disease *in vivo* (Brennan *et al.*, 1990; Seitz *et al.*, 1991; Meir *et al.*, 1992). Some IFH cells might produce GM-CSF and/or IL-8/NAP-1 spontaneously in the clinical setting. IL-1 was detected in some neoplastics such as Hodgkin's disease and histiocytic malignancy *in vivo* (Hsu & Zhao, 1986). Another possibility is that MFH cells may be stimulated by IL-1β secreted from themselves or accessory cells, and then produce GM-CSF and/or IL-8/NAP-1.

The potency of neutrophil chemotactic factor production by IFH cell lines might suggest a new approach to investigation of the pathological and clinical features in this disease.

This work was supported in part by Grant-in-Aid for Cancer Research (61–2) from the Ministry of Health and Welfare, and a grant (01770902) from the Ministry of Education, Science, and Culture of Japan.

### References

BRENNAN, F.M., ZACHARIAE, C.O.C., CHANTRY, D., TURNER, M., MAINI, R.N., MATSUSHIMA, K. & FELDMANN, M. (1990). Detection of interleukin 8 biological activity in synovial fluids from patients with rehumatoid arthritis and production of interleukin 8 mRNA by isolated synovial cells. *Eur. J. Immunol.*, **20,** 2141–2144.

GHERADI, G., AMBROSI, S. & SCHERNI, P. (1985). Acute inflammation in malignant fibrous histiocytoma. *Arch. Pathol. Lab. Med.*, **109.** 8–9.

HARVATH, L., FALK, W. & LEONARD, E.J. (1980). Rapid quantitation of neutrophil chemotaxis: Use of a polyvinylpyrrolidone-free polycarbonate membrane in a multiwell assembly. *J. Immunol. Methods*, **37,** 39–45.

HSU, S-M. & ZUAO, X. (1986). Expression of interleukin-1 in Reed-Sternberg cells and neoplastic cells from true histiocytic malignancies. *Am. J. Pathol.*, **125,** 221–225.

ISODA, M. & YASUMOTO, S. (1986). Eosinophil chemotactic factor derived from a malignant fibrous histiocytoma. *Clin. Exp. Dermatol.*, **11,** 253.

KYRIAKOS, M. & KEMPSON, R.L. (1976). Inflammatory fibrous histiocytoma: An aggressive and lethal lesion. *Cancer*, **37,** 1584–1606.

LE, J. & VILCEK, J. (1987). Biology of disease. Tumor necrosis factor and interleukin 1: Cytokines with multiple overlapping biological activities. *Lab. Invest.*, **56,** 234–248.

MATSUSHIMA, K., MORISHITA, K., YOSHIMURA, T., LAVU, S., KOBAYASHI, Y., LEW, W., APPELLA, E., KUNG, H.F., LEONARD, E.J. & OPPENHEIM, J.J. (1988). Molecular cloning of a human monocyte-derived neutrophil chemotactic factor (MDNCF) and the induction of MDNCSF mRNA by Interleukin 1 and tumor necrosis factor. *J. Exp. Med.*, **167,** 1883–1893.

MEIR, E.V., CESKA, M., EFFENBERGER, F., WALZ, A., GROUZMANN, E., DESBAILLETS, I., FREI, K., FONTANA, A. & DE TRIBOLET, N. (1992). Interleukin-8 is produced in neoplastic and infectious diseases of the human central nervous system. *Cancer Res.*, **52,** 4297–4305.

MUNKER, R., GASSON, J., OGAWA, M. & KOEFFLER, H.P. (1986). Recombinant human TNF induces production of granulocyte-monocyte colony-stimulating factor. *Nature*, **323,** 79–82.

OKABE, H., IMOKAWA, M., OCHI, Y., KOKUHO, M., HAYASHIDA, E., TOMOYOSHI, T. & TAKEOKA, O. (1987). A case of malignant fibrous histiocytoma: The use of tissue culture for identification of the histiocytic nature of the tumor cells. *J. Shiga Univ. Med. Sci.*, **2,** 105–110.

OPPENHEIM, J.J., KOVACS, E., MATSUSHIMA, K. & DURUM, S.K. (1986). There is more than one interleukin 1. *Immunol. Today*, **7**, 45–56.

ROQUES, A.W.W., HORTON, L.W.L., LESLIE, J. & BUXTON-THOMAS, M.S. (1979). Inflammatory fibrous histiocytoma in the left upper abdomen with a leukemoid blood picture. *Cancer*, **43**, 1800–1804.

SEITZ, M., DEWALD, B., GERBER, N. & BAGGIOLINI, M. (1991). Enhanced production of nutrophil-activating peptide-1/ interleukin-8 in rheumatoid arthritis. *J. Clin. Invest.*, **87**, 463–469.

SERKE, S., BRENNER, M., ZIMMERMANN, R. & LOBECK, H. (1986). Malignant fibrous histiocytoma associated with peripheral blood eosinophilia. *Oncology*, **43**, 230–233.

SHIRASUNA, K., SUGIYAMA, M. & MIYAZAKI, T. (1985). Establishment and characterization of neoplastic cells form a malignant fibrous histiocytoma: A possible stem cell line. *Cancer*, **55**, 2521–2532.

SOUTHERN, E.M. (1979). Detection of specific sequences among DNA fragments separated by gel electrophoresis. *J. Mol. Biol.*, **98**, 503–517.

STRIETER, R.M., KUNKEL, S.L., SHOWELL, H.J., REMICK, D.G., PHAN, S.H., WARD, P.A., MARKS, R.M. (1989). Endothelial cell gene expresseion of a neutrophil chemotactic factor by TNF-$\alpha$, LPS, and IL-1$\beta$. *Science*, **243**, 1467–1469.

TAKAHASHI, K., KIMURA, Y., NAITO, M., YOSHIMURA, T., UCHIDA, H. & ARAKI, S. (1989). Inflammatory fibrous histiocytoma presenting leukemoid reaction. *Path. Res. Pract.*, **184**, 498–506.

TAKEYA, M., YOSHIMURA, T., LEONARD, E.J., KATO, T., OKABE, H. & TAKAHASHI, K. (1991). Production of monocyte chemoattractant protein-1 by malignant fibrous histiocytoma: Relation to the origin of histiocyte-like cells. *Exp. Mol. Pathol.*, **54**, 61–71.

TANI, K., OZAWA, K., OGURA, H., SHIMANE, M., SHIRAFUJI, N., TSURUTA, T., YOKOTA, J., NAGATA, S., UEYAMA, Y., TAKAKU, F. & ASANO, S. (1990). Expression of granulocyte and granulocyte-macrophage colony-stimulating factors by human non-hematopoietic tumor cells. *Growth Factors*, **3**, 325–331.

WANG, J.M., COLELLA, S., ALLAVENA, P. & MANTOVANI, A. (1987). Chemotactic activity of human recombinant granulocyte-macrophage colony-stimulating factor. *Immunology*, **60**, 439–444.

WEISS, S.W. & ENZINGER, F.M. (1978). Malignant fibrous histiocytoma: An Analysis of 200 cases. *Cancer*, **41**, 2250–2260.

WONG, G.G., WITEK, J.S., TEMPLE, P.A., WILKENS, K.M., LEARY, A.C., LUXENBERG, D.P., JONES, S.S., BROWN, E.L., KAY, R.M., ORR, E.C., SHOEMAKER, C., GOLDE, D.W., KAUFMAN, R.J., HEWICK, R.M., WANG, E.A. & CLARK, S.C. (1985). Human GM-CSF: Molecular cloning of the complementary DNA and purification of the natural and recombinant proteins. *Science*, **228**, 810–815.

YOSHIDA, M., MATSUZAKI, H., SAKATA, K., TAKEYA, M.K., KATO, K., MIZUSHIMA, S., KAWAKITA, M. & TAKATSUKI, K. (1992). Neutrophil chemotactic factors produced by a cell line from thyroid carcinoma. *Cancer Res.*, **52**, 464–469.

YOSHIMURA, T., MATSUSHITA, K., OPPENHEIM, J.J. & LEONARD, E.J. (1987). Neutrophil chemotactic factor produced by lipopolysaccharide (LPS)-stimulated human blood mononuclear leukocytes: Partial characterization and separation from interleukin 1 (IL-1). *J. Immunol.*, **139**, 788–793.

ZUCALI, J.R., DINARELLO, C.A., OBLON, D.J., GROSS, M.A., ANDERSON, L. & WEINER, R.S. (1986). Interleukin 1 stimulates fibroblasts to produce granulocyte-macrophage colony-stimulating activity and prostaglandin $E_2$. *J. Clin. Invest.*, **77**, 1857–1863.

ZOJA, C., WANG, J.M., BETTONI, S., SIRONI, M. & RENZI, D. (1991). Interleukin-1$\beta$ and tumor necorsis factor-$\alpha$ induce gene expression and production of leukocyte chemotactic factors, colony-stimulating factors, and interleukin-6 in human mesangial cells. *Am. J. Pathol.*, **138**, 991–1003.